# Prediction of extubation outcome in critically ill patients: a systematic review and meta-analysis

**DOI:** 10.1186/s13054-021-03802-3

**Published:** 2021-11-15

**Authors:** Flavia Torrini, Ségolène Gendreau, Johanna Morel, Guillaume Carteaux, Arnaud W. Thille, Massimo Antonelli, Armand Mekontso Dessap

**Affiliations:** 1 Service de Médecine Intensive Réanimation, Hôpitaux Universitaires Henri Mondor, AP-HP, 1 rue Gustave Eiffel, 94010 Créteil Cedex, France; 2 CARMAS, Univ Paris Est Créteil, 94010 Créteil, France; 3 INSERM, IMRB, Univ Paris Est Créteil, 94010 Créteil, France; 4 Centre d’Investigation Clinique 1402 ALIVE, Université de Poitiers, Poitiers, France; 5 Service de Médecine Intensive Réanimation, Centre Hospitalier Universitaire de Poitiers, Poitiers, France; 6 Fondazione Policlinico Universitario A. Gemelli IRCCS, Rome, Italy

**Keywords:** Airway extubation, Ventilator weaning, Risk factors

## Abstract

**Background:**

Extubation failure is an important issue in ventilated patients and its risk factors remain a matter of research. We conducted a systematic review and meta-analysis to explore factors associated with extubation failure in ventilated patients who passed a spontaneous breathing trial and underwent planned extubation. This systematic review was registered in PROPERO with the Registration ID CRD42019137003.

**Methods:**

We searched the PubMed, Web of Science and Cochrane Controlled Register of Trials for studies published from January 1998 to December 2018. We included observational studies involving risk factors associated with extubation failure in adult intensive care unit patients who underwent invasive mechanical ventilation. Two authors independently extracted data and assessed the validity of included studies.

**Results:**

Sixty-seven studies (involving 26,847 participants) met the inclusion criteria and were included in our meta-analysis. We analyzed 49 variables and, among them, we identified 26 factors significantly associated with extubation failure. Risk factors were distributed into three domains (comorbidities, acute disease severity and characteristics at time of extubation) involving mainly three functions (circulatory, respiratory and neurological). Among these, the physiological respiratory characteristics at time of extubation were the most represented. The individual topic of secretion management was the one with the largest number of variables. By Bayesian multivariable meta-analysis, twelve factors were significantly associated with extubation failure: age, history of cardiac disease, history of respiratory disease, Simplified Acute Physiologic Score II score, pneumonia, duration of mechanical ventilation, heart rate, Rapid Shallow Breathing Index, negative inspiratory force, lower PaO_2_/FiO_2_ ratio, lower hemoglobin level and lower Glasgow Coma Scale before extubation, with the latest factor having the strongest association with extubation outcome.

**Conclusions:**

Numerous factors are associated with extubation failure in critically ill patients who have passed a spontaneous breathing trial. Robust multiparametric clinical scores and/or artificial intelligence algorithms should be tested based on the selected independent variables in order to improve the prediction of extubation outcome in the clinical scenario.

**Supplementary Information:**

The online version contains supplementary material available at 10.1186/s13054-021-03802-3.

## Background

As mechanical ventilation is associated with complications (e.g., ventilator-associated pneumonia) [1], the optimal time to wean patients from mechanical ventilation is a critical goal to achieve in intensive care unit (ICU) [2]. The decision to extubate is therefore usually taken as soon as a patient meet predefined weaning criteria and successfully pass a spontaneous breathing trial (SBT) [3]. Nevertheless, in 10–20% of patients who pass a spontaneous breathing trial and undergo planned extubation, extubation failure still occurs.

Extubation failure is usually defined as the need for reintubation within hours or days after a planned extubation. The time considered varies from 24 h [4, 5] until any time during the hospital stay [6, 7]. Extubation failure is associated with an overall increase in the duration of mechanical ventilation, a greater need for tracheostomy, higher medical costs and a 25–50% increased mortality rate [8–12]. There is some evidence that extubation failure is not just a marker of a more severe illness, but independently affects patients survival regardless of underlying illness severity [9, 13].

Unfortunately, the pathophysiology of extubation failure is not yet fully understood and a simple tool for predicting extubation failure is not available. Some studies suggested that the use of standardized weaning protocols reduced the total time of mechanical ventilation [14, 15] but the parameters that should be included in weaning protocols still remain controversial. Considering the complications associated with both a too early and delayed liberation from mechanical ventilation, the identification of robust risk factors for extubation failure would be extremely helpful in order to optimize the weaning process.

We therefore decided to conduct a systematic review of the literature and a meta-analysis to search risk factors associated with extubation failure, in adult critically ill patients who passed a SBT and underwent a planned extubation.

## Methods

### Search strategy and selection criteria

We performed this study in accordance with the Preferred Reporting Items for Systematic Reviews and Meta-Analyses (PRISMA) statement [16]. We searched PubMed, Web of Science and Cochrane controlled register of trials (CENTRAL) to identify articles on risk factors for extubation failure from January 1998 to December 2018. We used the following search algorithm: (extubation) AND (success OR failure OR factor OR predictor OR prediction OR risk OR score OR outcome OR mortality OR reintubation OR intensive care unit).

We included all studies that evaluated any risk factors for extubation failure in adult (at least 18 years old) ICU patients under invasive mechanical ventilation. We excluded studies in children and animals and studies not written in English. References of all selected articles were scanned for additional relevant manuscripts. This study was registered in the International Prospective Register of Systematic Reviews (PROSPERO) database (Registration Number CRD42019137003). Ethical approval was not required.

### Data analysis

After the removal of the duplicates, two authors (FT, JM) independently screened titles and abstracts to obtain relevant articles for full text review. We obtained the full text of all potentially relevant studies and the authors independently decided for final inclusion in the review. We also reviewed the references of relevant articles to avoid missing any studies. Any disagreement was resolved by consensus or discussion with a third reviewer (AMD).

The review authors independently extracted data. The following data were recorded from each selected study: year of publication, study design, baseline characteristics of the population (age, comorbidities), severity scores on ICU admission and stay [Severity Acute Physiologic Score (SAPS), Acute Physiology and Chronic Health Evaluation (APACHE)], medications, characteristics of the SBT, definition of extubation failure, risk factors associated with extubation failure (respiratory, cardiovascular, neurologic, laboratory parameters) and primary outcome (extubation failure). We further excluded risk factors with excessive missing data (reported in less than 10% of studies; see Additional file 1: Table S1 in the additional material). Study quality was assessed in terms of risk of bias using the QUIPS tool for prognostic studies (Cochrane), rating the potential risk of bias as high, moderate or low for each of six domains, namely study participation, study attrition, prognostic factor measurement, outcome measurement, study confounding, statistical analysis and reporting. Two authors (FT, JM) independently assessed the risk of bias, implying a third author in case of disagreement (AMD).

### Statistical analysis

We conducted a meta-analysis of observational prospective and retrospective studies. Data were summarized using medians and interquartile ranges (IQRs) or mean ± standard deviation (SD) were appropriate [17]. For binary variables, the odds ratio (OR) with 95% confidence interval (CI) was calculated for extubation failure. For continuous variables, we calculated the mean difference with 95% CI between extubation success and extubation failure groups. A natural log transformation of OR (lnOR) was derived from crude OR (for binary variables) [18, 19] and from standardized mean differences (for continuous variables) [19] in a symmetric scale, from minus infinity, to infinity, with zero defining no effect [18], to allow comparison between categorical and numeric variables.

We adopted the inverse variance method for developing weights for individual study effects. We quantified heterogeneity using *I*^2^ and *Q* statistics, with values greater than 50% regarded as being indicative of moderate-to-high heterogeneity [20]. We used a random effect model to assess the population average mean difference and 95% CI or OR and 95% CI for all the risk factors for extubation failure. In order to measure the dispersion of the pooled effect across study settings, we generated predictions intervals [21].

We performed prespecified subgroup analyses according to the type of ICU patients, e.g., medical, surgical, mixed, neurological or other type of ICU. A heatmap was created to present lnOR (scaled to adjust for extreme values) for each variable according to ICU type. We conducted a sensitivity analysis including only studies referring to the most used definition of extubation failure (death or reintubation within 48 h from extubation), to explore if it changed the significance of the results. Another sensitivity analysis focused on studies referring to death or reintubation (whatever the delay).

A multivariable meta-analysis of multiple factors was secondarily performed with variables significantly associated with extubation outcome, using effect sizes as lnOR [22] and the *altmeta* package for R [23, 24]. Among related significant univariate factors, only the most statistically robust (as per the lnOR), yet clinically relevant were entered into the models in order to minimize the effect of collinearity. Individual study effects and pooled effects were visualized through forest plots.

Publication biases were assessed graphically through funnel plot asymmetry [25]. Data were pooled and analyzed using Review Manager (Cochrane TC. Review Manager 5.3. Cph Nord Cochrane Cent, 2008) with a two-sided significance level of 5%, and R 3.1.2 (The R Foundation for Statistical Computing, Vienna, Austria).

## Results

### Studies

**Fig. 1:**
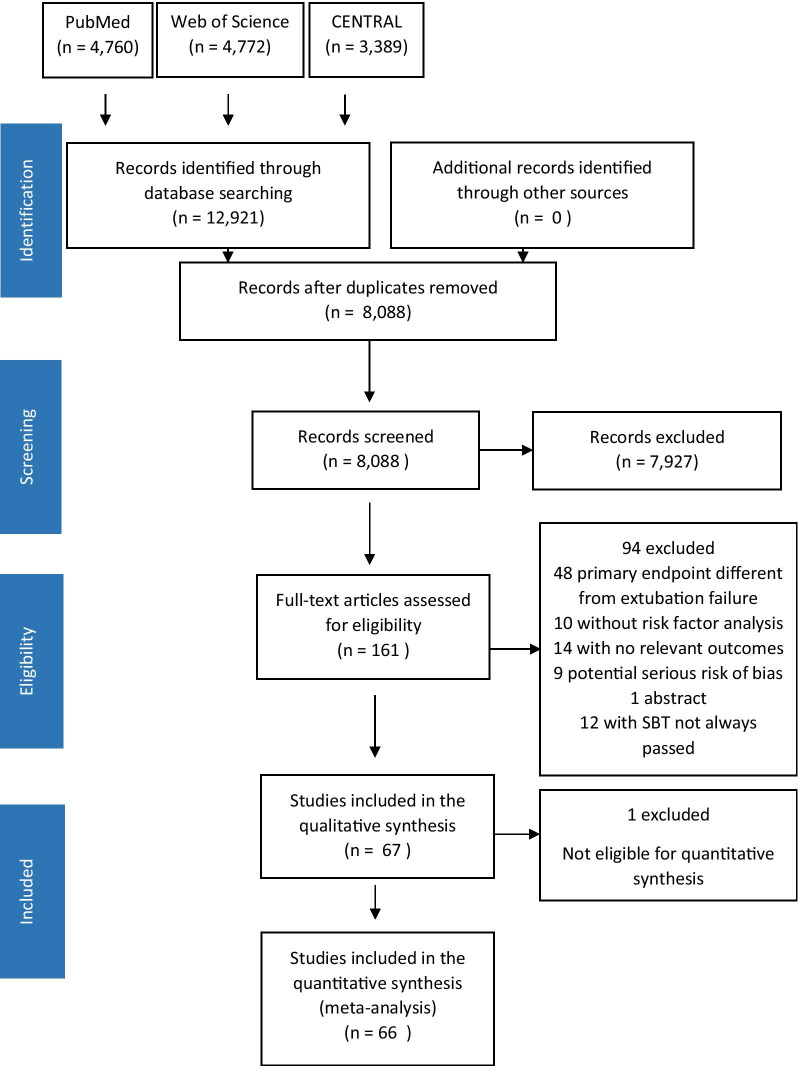
Study flowchart

We identified a total of 12,921 references from our searches (Fig. 1). After removing 4833 duplicates, we screened the titles and abstracts of 8088 articles, of which 7927 were excluded. The full texts of 161 studies were reviewed and 94 were excluded. Thus, we included 67 studies in the narrative review, and 66 studies were included in the quantitative synthesis. Of the 67 studies included in the review, 50 were prospective observational studies [4–6, 9, 12, 13, 26–69] and 17 were retrospective studies [7, 70–85]. Fifty-seven studies were monocentric and ten were multicentric. Studies took place in medical (*n* = 19), surgical (*n* = 9), mixed (*n* = 28) and neurological (*n* = 11) ICUs.

We included 67 studies involving 26,847 participants in the meta-analysis. The type of SBT and the definition of extubation failure varied among the included studies (Table 1). SBT types included: multiple choice for SBT (*n* = 15, with 4 studies allowing CPAP and 14 studies allowing T-tube), low pressure support with low positive end expiratory pressure (*n* = 30), flow-by (*n* = 1), standard pressure support ventilation (*n* = 1), proportional assist ventilation (*n* = 1) and automatic tube compensation with low PEEP (*n* = 1). Extubation failure was defined either as death or reintubation within hours to days after extubation (24 h, 48 h, 72 h or 7 days in 4, 30, 14 and 5 studies, respectively), or as the reinstitution of any mechanical ventilation after extubation, either invasive or noninvasive with a curative indication (10 studies).

**Table 1:** Type of spontaneous breathing trial and definition of extubation failure

Variable	Number of studies (%)
*Spontaneous breathing trial*	
T piece	15 (22.4%)
Low pressure support with zero-end expiratory pressure	6 (9.0%)
Continuous positive airway pressure	3 (4.5%)
Other	33 (49.3%)
Not available	10 (14.8%)
*Duration of SBT*	
30 min	12 (17.9%)
60 min	8 (11.9%)
120 min	13 (19.4%)
Variable*	21 (31.3%)
Not available	12 (17.9%)
*Definition of SBT failure*	
Respiratory rate	43 (64.2%)
Increased breathing work or distress signs	31 (46.9%)
Arterial oxygen saturation	39 (58.2%)
PaO2	8 (11.9%)
PaCO2	10 (14.9%)
Tidal volume or minute ventilation or RSBI	11 (16.4%)
Heart rate	36 (53.7%)
Arterial pressure or introduction of vasopressive drug	36 (53.7%)
Neurological criteria	38 (56.7%)
Not available	20 (29.9%)
*Definition of extubation failure*	
Death or reintubation	
Within 24 h	4 (6.0%)
Within 48 h	30 (44.8%)
Within 72 h	14 (20.9%)
Within 7 days	5 (7.5%)
At any time until discharge or death	4 (6.0%)
Reintubation or curative non-invasive mechanical ventilation	10 (14.9%)

*SBT* spontaneous breathing trial, *PaO2* partial pressure of oxygen, *PaCO2* partial pressure of carbon dioxide, *RSBI* Rapid Shallow Breathing Index, * from 30 min to 12 h

### Risk factors for extubation failure

**Fig. 2:**
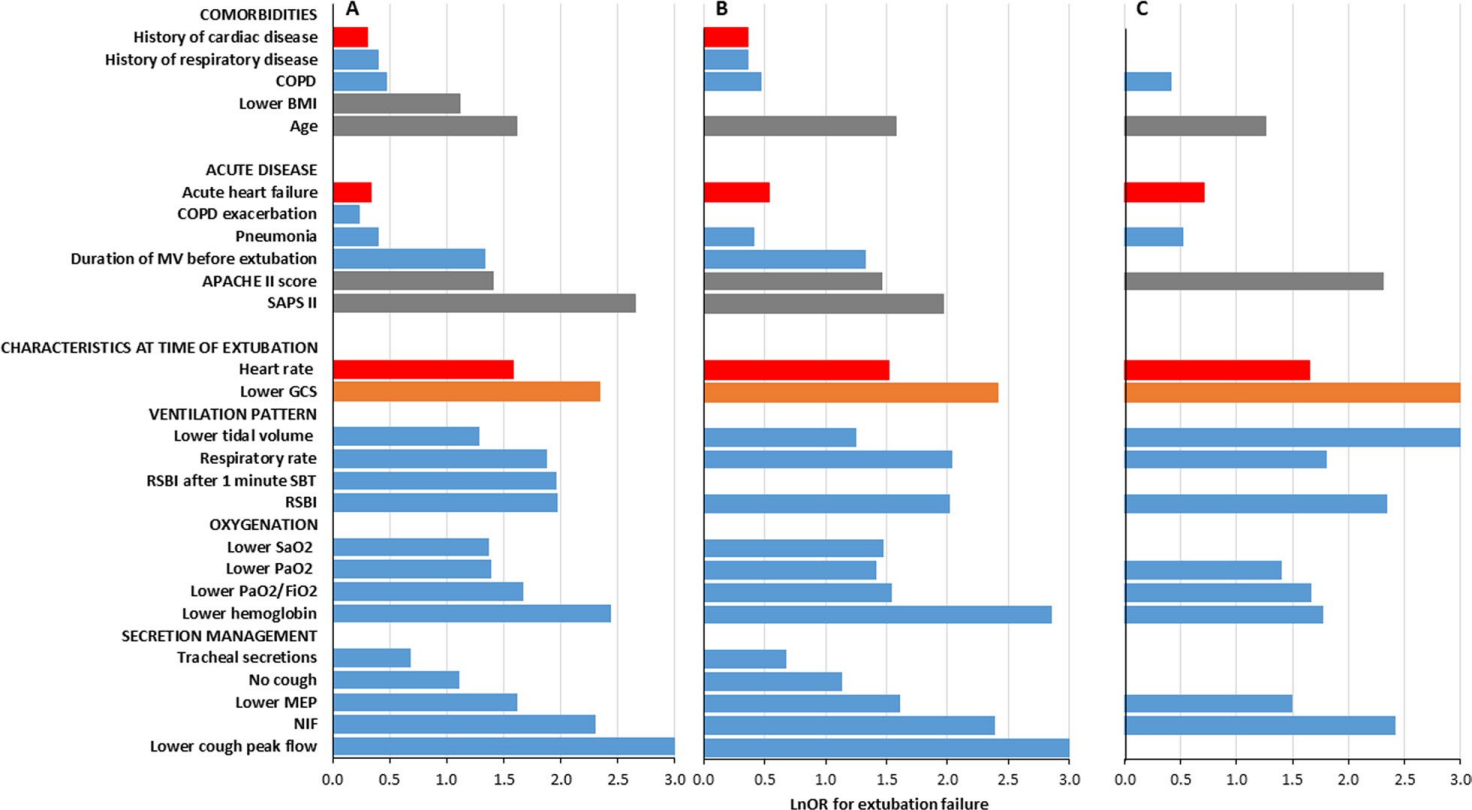
Natural log transformation of odd ratios summarizing variables significantly associated with extubation outcome. **A** Overall analysis; **B** Sensitivity analysis focusing on studies defining extubation failure as death or reintubation, whatever the delay; **C** Sensitivity analysis focusing on studies defining extubation failure at 48 h. Natural log transformation of odd ratios (lnOR) were derived from crude OR (for binary variables) and from standardized mean differences (for continuous variables) to summarize the effect of 26 variables significantly associated with extubation outcome, involving three main functions [respiratory (blue bars), circulatory (red bars), neurological (orange bars) and scores/physiological data (grey bars)]. COPD: chronic obstructive pulmonary disease; BMI: body mass index; GCS: Glasgow coma scale; NIF: negative inspiratory force; SAPSII: simplified acute physiology score; RSBI: rapid shallow breathing index; SBT: spontaneous breathing trial; MEP: maximal expiratory pressure; MV: mechanical ventilation

Among the 49 variables analyzed, we found 26 variables significantly associated with extubation outcome, distributed into three domains [comorbidities (*n* = 5), acute disease severity (*n* = 6) and characteristics at time of extubation (*n* = 15)] (Table 2, Fig. 2) involving mainly three functions [respiratory (*n* = 16), circulatory (*n* = 3) and neurological (*n* = 1)]; see Additional file 1 Fig. S1 in the additional material).

**Table 2:** Variables analyzed in the meta-analysis

Variables	*n*	*N*	Statistical method	Effect estimate [95%CI]
Age*	59	23,426	Mean difference	3.43 [2.44, 4.41]
APACHE II score*	33	15,696	Mean difference	1.63 [0.92, 2.35]
Body mass index*	13	8483	Mean difference	− 0.64 [− 1.21, − 0.08]
Male sex	52	22,093	Odds ratio	0.90 [0.76, 1.07]
SAPS II*	15	7159	Mean difference	4.20 [2.75, 5.65]
History of cardiac disease*	16	7298	Odds ratio	1.35 [1.12, 1.64]
History of respiratory disease*	11	6303	Odds ratio	1.49 [1.18, 1.87]
COPD*	12	1984	Odds ratio	1.60 [1.16, 2.21]
Acute heart failure*	11	3947	Odds ratio	1.40 [1.04, 1.89]
ARDS	9	2842	Odds ratio	1.13 [0.75, 1.69]
COPD exacerbation*	18	4183	Odds ratio	1.26 [1.01, 1.58]
Glasgow Coma Scale before ext.*	13	5933	Mean difference	− 0.75 [− 1.06, − 0.43]
Pneumonia*	17	3692	Odds ratio	1.48 [1.21, 1.81]
Albumin	9	5481	Mean difference	− 0.21 [− 0.43, 0.02]
Hemoglobin*	18	7277	Mean difference	− 0.54 [− 0.72, − 0.35]
PaCO_2_ before ext	34	12,328	Mean difference	0.81 [− 0.02, 1.64]
PaO_2_ before ext.*	22	9677	Mean difference	− 8.02 [− 12.39, − 3.66]
PaO_2_/FiO_2_ before ext.*	30	11,960	Mean difference	− 19.38 [− 26.92, − 11.84]
SaO_2_ before ext.*	7	1893	Mean difference	− 0.44 [− 0.87, − 0.01]
Duration of MV before ext.*	46	19,775	Mean difference	1.03 [0.62, 1.43]
Respiratory rate before ext.*	27	15,178	Mean difference	1.86 [1.19, 2.54]
RSBI*	44	20,301	Mean difference	8.51 [6.20, 10.81]
RSBI after 1 min SBT*	8	1606	Mean difference	10.26 [3.68, 16.84]
Tidal volume before ext.*	25	12,070	Mean difference	− 28.69 [− 44.61, − 12.78]
Heart rate before ext.*	20	9848	Mean difference	2.99 [1.49, 4.49]
Maximal expiratory pressure*	9	12,183	Mean difference	− 10.22 [− 17.70, − 2.73]
Negative inspiratory force*	14	13,448	Mean difference	5.30 [3.11, 7.48]
Cough*	7	3337	Odds ratio	0.33 [0.16, 0.66]
Cough peak flow*	8	1041	Mean difference	− 27.50 [− 38.95, − 16.04]
Moderate/abundant secretions*	7	2248	Odds ratio	1.98 [1.14, 3.43]
Acute respiratory failure	7	1249	Odds ratio	1.43 [0.88, 2.32]
Coma	8	2742	Odds ratio	0.77 [0.57, 1.03]
Creatinine	9	5422	Mean difference	0.11 [− 0.06, 0.29]
Diastolic blood pressure before ext	9	1651	Mean difference	− 1.03 [− 2.57, 0.50]
Diabetes	15	5976	Odds ratio	1.27 [0.96, 1.69]
FiO_2_ during SBT	11	7818	Mean difference	0.00 [− 0.00, 0.01]
Glasgow Coma Scale upon admission	14	9113	Mean difference	− 0.28 [− 0.57, 0.00]
History of hypertension	8	998	Odds ratio	1.09 [0.78, 1.52]
Mean arterial pressure before ext	7	5161	Mean difference	− 0.95 [− 2.36, 0.45]
Minute ventilation before ext	17	14,383	Mean difference	0.00 [− 0.34, 0.34]
Neurologic diagnosis	9	3357	Odds ratio	1.19 [0.76, 1.87]
PEEP during SBT	11	7214	Mean difference	0.05 [− 0.00, 0.10]
pH before ext	27	11,392	Mean difference	− 0.00 [− 0.01, 0.00]
Postoperative respiratory failure	7	2713	Odds ratio	1.01 [0.69, 1.48]
SBP before ext	13	5240	Mean difference	− 0.41 [− 1.95, 1.13]
Sepsis	7	2903	Odds ratio	1.17 [0.92, 1.48]
Shock	8	1722	Odds ratio	0.87 [0.50, 1.50]
Steroids	7	3674	Odds ratio	0.84 [0.58, 1.24]
Trauma	7	4916	Odds ratio	0.83 [0.63, 1.09]

*N* number of participants, *n* number of studies, *ext* extubation, *SBT* spontaneous breathing trial, *SAPS* severity acute physiologic score, *APACHE* acute physiology and chronic health evaluation, *COPD* chronic obstructive pulmonary disease, *ARDS* acute respiratory distress syndrome, *PaCO2* partial pressure of carbon dioxide in the arterial blood, *PaO2* partial pressure of oxygen in the arterial blood, *SaO2* oxygen saturation in the arterial blood, *FiO2* fraction of inspired oxygen, *RSBI* Rapid Shallow Breathing Index, *PEEP* positive end expiratory pressure, *statistically significant by meta-analysis

#### Comorbidities

We found a higher risk of extubation failure in older patients. Chronic obstructive respiratory disease, history of chronic cardiac or respiratory disease were associated with a higher risk of extubation failure as well. In contrast, a higher body mass index was associated with successful extubation.

#### Acute disease severity

Patients who failed extubation also differed from patients who succeeded in terms of acute disease severity, with higher values of SAPS II and APACHE II score in the former group. Acute heart failure, COPD exacerbation and pneumonia were the reasons for intubation significantly associated with a higher risk of extubation failure. Duration of mechanical ventilation before extubation was longer in patients with extubation failure.

#### Characteristics at the time of extubation

These variables involved the following physiological systems: (1) respiratory: related to secretion management (cough, cough peak flow, maximal expiratory pressure, presence of moderate to abundant secretions, negative inspiratory force), ventilation pattern [respiratory rate and tidal volume before extubation, rapid shallow breathing index (RSBI) after one minute from the SBT start, RSBI before extubation] and oxygenation (SaO_2_, PaO_2_ and PaO_2_/FiO_2_ before extubation, hemoglobin on the day of extubation); (2) cardiovascular (heart rate before extubation); and (3) neurological (Glasgow Coma Scale before extubation). The individual topic of secretion management was the one with the largest number variables (five).

### Subgroup analysis

Subgroup analyses according to the type of ICU are presented in Additional file 1: Fig. S2 and Table S2 in the additional material. Among the 49 variables analyzed, 30 were significantly associated with extubation outcome in at least one ICU type. Eight factors were significant in the majority of ICU types (at least three among the five types), including age, SAPS II score, duration of mechanical ventilation before extubation, heart rate, respiratory rate, RSBI, PaO_2_ before extubation and cough peak flow. Duration of mechanical ventilation had the broadest association across ICU types (4/5 types), while cough peak flow had the strongest association across ICU types (Additional file 1: Fig. S2).

### Sensitivity analysis

Due to the heterogeneity among studies in terms of definition of extubation failure, we performed an exploratory sensitivity analysis restricted to studies where extubation failure was defined as death or reintubation, whatever the delay (57 out of 67 included in the meta-analysis): the vast majority of variables identified by crude analysis (23) remained significant, while only three were not (see Fig. 2, and Additional file 1: Table S3 in the additional material). Another sensitivity analysis was restricted to studies where extubation failure was defined as death or reintubation within 48 h (30 articles out of 67 included in the meta-analysis): 15 variables remained significant while eleven were not, including factors related to cough and tracheal secretions (see Fig. 2, and Additional file 1: Table S4 in the additional material).

### Multivariable analysis

**Fig. 3:**
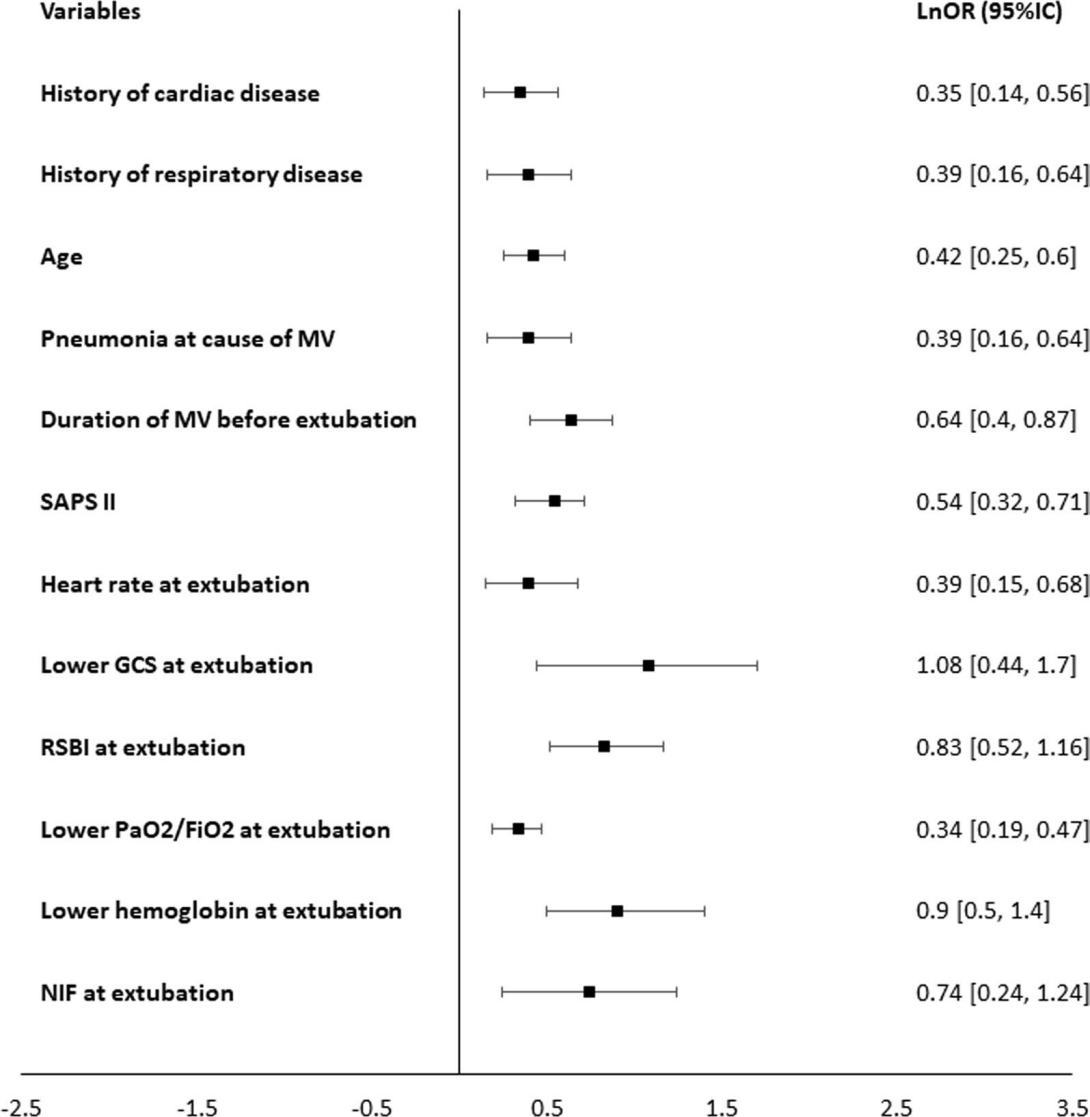
Forest plot for the twelve variables retained in the final model, significantly associated with extubation failure in multivariable meta-analysis. Effects are reported in natural log transformation of odd ratios (lnOR) derived from crude OR with 95% confidence interval margins (CI). NIF: negative inspiratory force; SAPSII: simplified acute physiology score; RSBI: rapid shallow breathing index; MV: mechanical ventilation

The 26 variables significantly associated with extubation outcome were assessed using a multivariable analysis for multiple factors (Additional file 1: Fig. S3 in the additional material). Twelve variables (age, history of cardiac disease, history of respiratory disease, SAPS II score, duration of mechanical ventilation, pneumonia, heart rate, RSBI, negative inspiratory force, lower PaO_2_/FiO_2_, lower Glasgow Coma Scale and lower hemoglobin level before extubation) were retained in the final model (Fig. 3). Glasgow Coma Scale (GCS) before extubation had the strongest independent association with extubation outcome.

### Quality

**Fig. 4:**
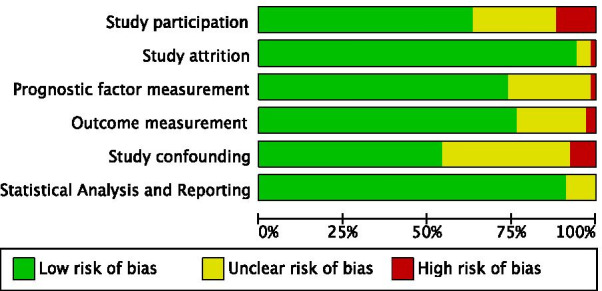
Summary of risk of bias in the included studies

Included studies differed in their methodological quality (Fig. 4, and Additional file 1: Fig. S4 in the additional material). High risk of bias was related to the study participation in eight studies [47, 57, 78–82, 85], to study attrition in one study [33], to prognostic factor measurement in one study [7], to the outcome measurement in two studies [29, 77] and to study confounding in five studies [27, 65, 78, 81, 85]. The remaining studies had low or unclear risk of bias for each of the six domains.

## Discussion

To the best of our knowledge, we herein report the first meta-analysis on factors associated with extubation failure. By multivariable analysis, twelve factors were significantly associated with extubation failure: age, history of cardiac disease, history of respiratory disease, SAPS II score, duration of mechanical ventilation, pneumonia, heart rate, RSBI, negative inspiratory force, lower PaO_2_/FiO_2_, lower hemoglobin level before extubation and lower Glasgow Coma Scale before extubation, with the latest factor having the strongest independent association with extubation outcome.

### Definition of extubation failure

An important information that comes out from our systematic review is that there is lack of standardization about the definition of extubation failure. It was defined as death or reintubation within a time interval that varies from 24 h to 7 days and in some studies, it also comprised the need for curative noninvasive ventilation after extubation. This leads to a risk of bias in evaluating the prognostic factors for extubation failure. For this reason, we performed a sensitivity analysis considering only studies where extubation failure was defined as death or reintubation within 48 h, since this is the most used definition. In this sensitivity analysis, the majority (15 out of 26) of factors identified by the crude analysis remained significant. However, cough, cough peak flow and secretions were no longer associated with extubation outcome when considering a 48 h delay. Alteration of cough and/or excessive secretions may act as major cause of delayed reintubation. These findings, and the increasing use of prophylactic non-invasive ventilation and high flow oxygen after extubation, may suggest the use of death or reintubation within a seven-day delay to define extubation failure in future studies [86].

### Risk factors for extubation failure

As highlighted in the present work, many factors may contribute to extubation failure in a given critically ill patient, suggesting an individual pathophysiological approach. The topic of secretion management was the one with the largest number of variables (five) significantly associated with extubation failure, pointing out that it should be carefully evaluated before extubation. The assessment of the “upper airway patency”, in terms of amount of secretions and the ability to clear them through an effective cough, has been increasingly used in the literature, even though these parameters are difficult to measure in an objective and standardized way. Cough peak flow is a parameter that has been proposed in the last few years to overcome this problem, but our multivariable analysis suggests negative inspiratory pressure as a relevant indicator.

Although the majority of statistically significant variables from our meta-analysis were related to the respiratory function (16 variables), the circulatory (three variables) and neurological (one variable) functions were also involved, with Glasgow Coma Scale having the strongest association with extubation outcome by multivariable analysis. These results are consistent with the plurality of often-intertwined mechanisms of extubation failure. Thus, restricting the clinical reasoning to the spectrum of a few variables related to the respiratory function may weaken the decisional process of liberation from mechanical ventilation. Robust multiparametric clinical scores and/or artificial intelligence algorithms should be tested based on the selected variables in order to improve the prediction of extubation outcome in the clinical scenario.

### Strengths and limitations

Strengths of our study include the wide period of assessment and selection process. One limitation is the lack of standardization in the definition of extubation failure. However, we performed a sensitivity analysis using the most accepted definition. Another limitation is that, due to the lack of data, we could not analyze the postextubation stridor, which is considered a rare but important risk factor for extubation failure. Finally, we may have missed other potentially important factors since we chose to analyze only parameters evaluated in at least 10% of included studies.

## Conclusion

We performed a systematic review and meta-analysis of a wide population of critically ill patients, finding 26 and 12 risk factors for extubation failure in patients who have successfully passed a spontaneous breathing trial by univariate and multivariable analysis, respectively. These factors were related to age, comorbidities, acute disease severity and physiological characteristics at the time of extubation. To further explore these factors and their combination, a unique definition of extubation failure is needed. An automated algorithm incorporating these factors would probably be very useful to inform the decisional process of extubation.

## Supplementary Information

**Additional file 1: Table S1.** Variables excluded because of missing data. **Figure S1.** Forest plots for variables statistically significantly associated with extubation failure. **Table S2.** Subgroup analyses by intensive care unit type. **Figure S2.** Heatmap of natural log transformation of odd ratios (LnOR) for extubation failure, according to ICU type. **Table S3.** Sensitivity analysis focusing on studies defining extubation failure as death or reintubation, whatever the delay. **Table S4.** Sensitivity analysis focusing on studies defining extubation failure at 48 h. **Figure S3.** Individual risk of bias in the included studies. **Figure S4.** Forest plot for the 26 variables significantly associated with extubation failure, assessed by multivariate meta-analysis for multiple factors.

## Data Availability

Data sharing is not applicable to this article as no datasets were generated or analyzed during the current study.

## Abbreviations

APACHE: Acute physiology and chronic health evaluation
ARDS: Acute respiratory distress syndrome
BMI: Body mass index
CI: Confidence interval
COPD: Chronic Obstructive Pulmonary Disease
FiO2: Fraction of inspired oxygen
GCS: Glasgow Coma Scale
ICU: Intensive care unit
IQRs: Interquartile ranges
MEP: Maximal expiratory pressure
MV: Mechanical ventilation
NIF: Negative inspiratory force
OR: Odds ratio
PaCO2: Partial pressure of carbon dioxide in the arterial blood
PaO2: Partial pressure of oxygen in the arterial blood
PEEP: Positive end expiratory pressure
RSBI: Rapid shallow breathing index
SaO2: Oxygen saturation in the arterial blood
SAPS: Severity acute physiologic score
SBT: Spontaneous breathing trial
SD: Standard deviation
