# Supplementary material for: Prediction of extubation outcome in critically ill patients: a systematic review and meta-analysis
Source: Crit Care. 2021 Nov 15;25:391. doi: 10.1186/s13054-021-03802-3 (PMC8591441; doi:10.1186/s13054-021-03802-3)

# Prediction of extubation outcome in critically ill patients: a systematic review and meta-analysis.

**Additional file**

Flavia TORRINI, Ségolène GENDREAU, Johanna MOREL, Guillaume CARTEAUX, Arnaud W THILLE, Massimo ANTONELLI, Armand MEKONTSO DESSAP

**Additional table S1:** Variables excluded because of missing data.

| Variable tested | Number (%) of studies |  |
| --- | --- | --- |
| Arrhythmia | 1 (1%) |  |
| Aspiration | 1 (1%) |  |
| Atelectasis | 2 (3%) |  |
| Burns | 1 (1%) |  |
| Deep vein thrombosis | 1 (1%) |  |
| Delirium tremens | 1 (1%) |  |
| Difficult intubation | 0 (0%) |  |
| Hospital cost (NT$10,000) | 1 (1%) |  |
| Hospital mortality | 4 (6%) |  |
| Hospital stay (days) | 8 (12%) |  |
| Baseline IAP, mmH20 | 1 (1%) |  |
| IAP, mmH2O | 1 (1%) |  |
| ICU mortality | 2 (3%) |  |
| ICU stay (days) | 6 (9%) |  |
| ICU LOS prior to first SBT, days | 1 (1%) |  |
| ICU LOS/% burn | 1 (1%) |  |
| Modified Rankin scale | 1 (1%) |  |
| Mortality at 28 days | 3 (4%) |  |
| Mortality at 90 days | 1 (1%) |  |
| Multiple antibiotics (>3) | 1 (1%) |  |
| Myocardial infarction | 1 (1%) |  |
| Number of intubation | 1 (1%) |  |
| Pleural effusion | 1 (1%) |  |
| Pneumothorax | 1 (1%) |  |
| Postextubation hospital stay | 1 (1%) |  |
| Postextubation ICU stay | 2 (3%) |  |
| Pulmonary complications | 1 (1%) |  |
| Pulmonary embolism | 1 (1%) |  |
| Reoperation – n (%) | 1 (1%) |  |
| Rhabdomyolysis | 1 (1%) |  |
| Total duration of MV, days | 3 (4%) |  |
| Tracheostomy | 1 (1%) |  |
| Transferred to respiratory care center | 1 (1%) |  |
| Transferred to respiratory care ward | 1 (1%) | |
| Ventilator-associated pneumonia | 2 (3%) |  |

IAP: Intra-abdominal pressure; ICU: intensive-care unit; LOS: level of service; SBT: spontaneous breathing trial

**Additional figure S1.** Forest plots for variables statistically significantly associated with extubation failure.

1. Acute Heart failure


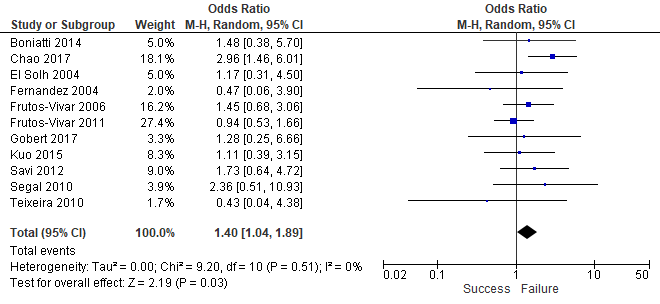


1. Age


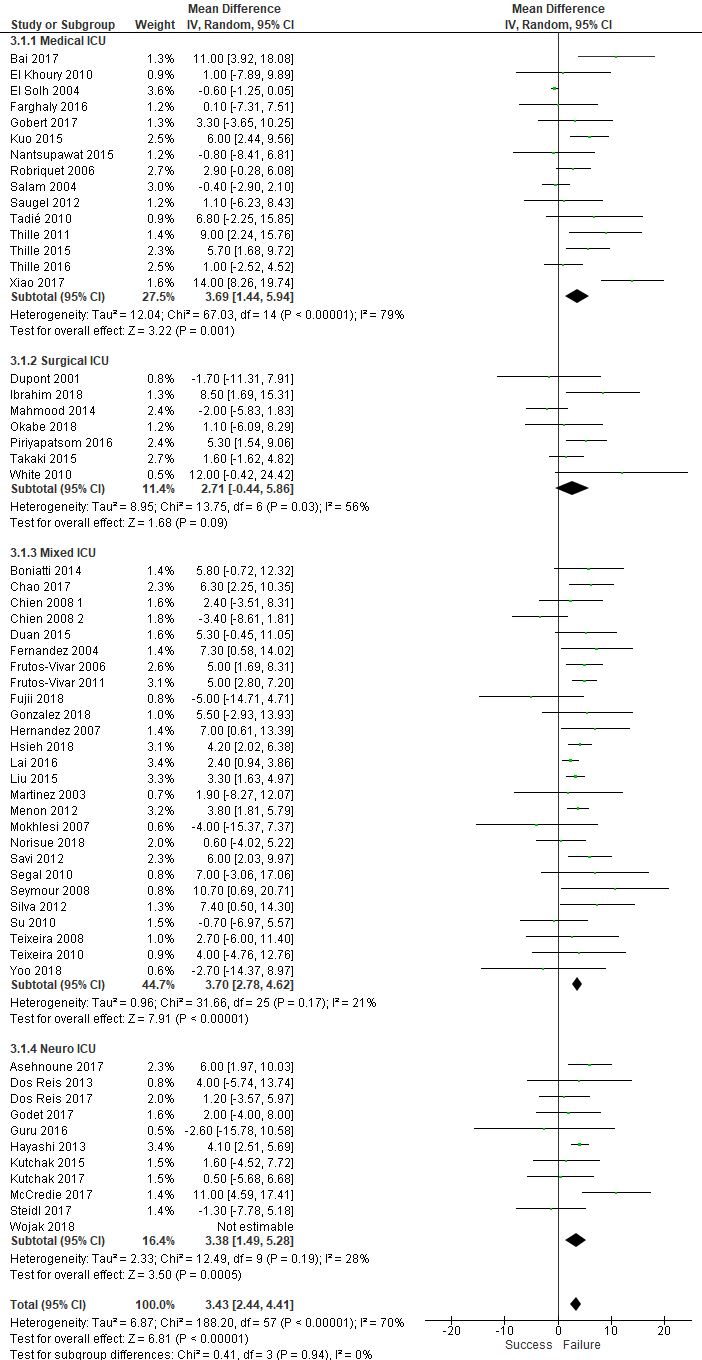


1. APACHE II score


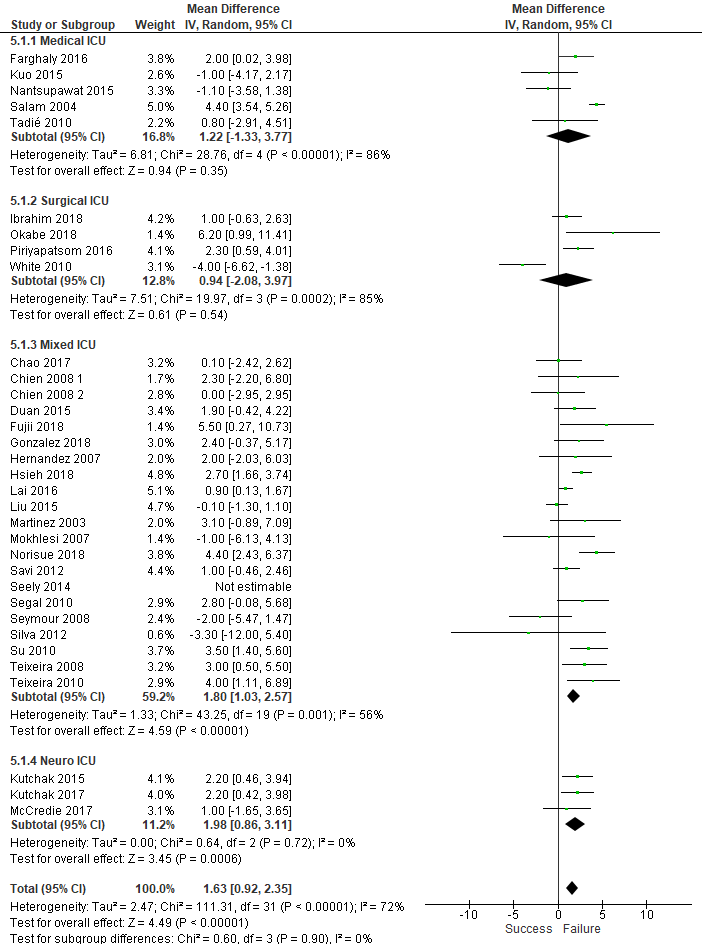


1. BMI


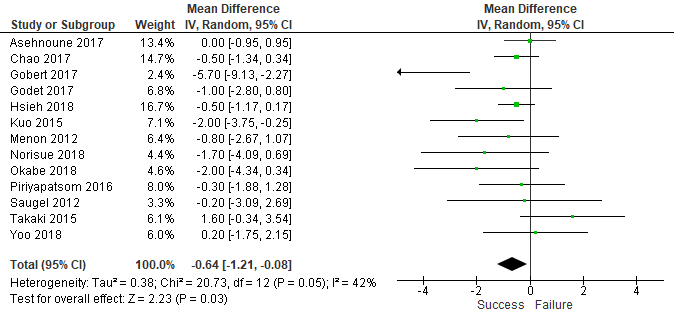


1. COPD


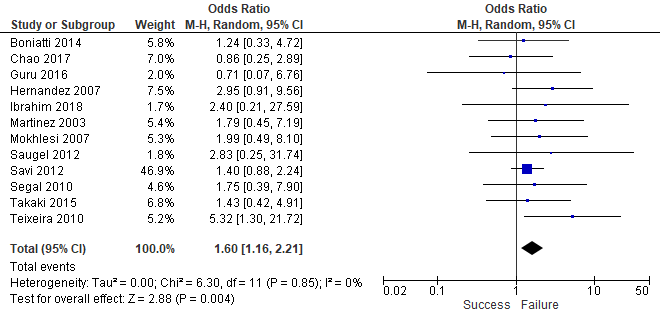


1. COPD exacerbation


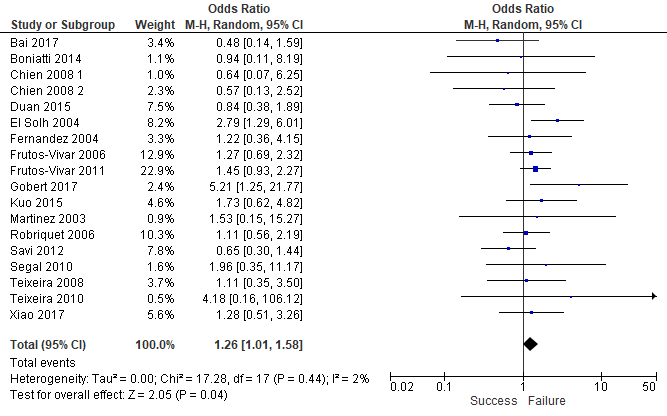


1. Cough


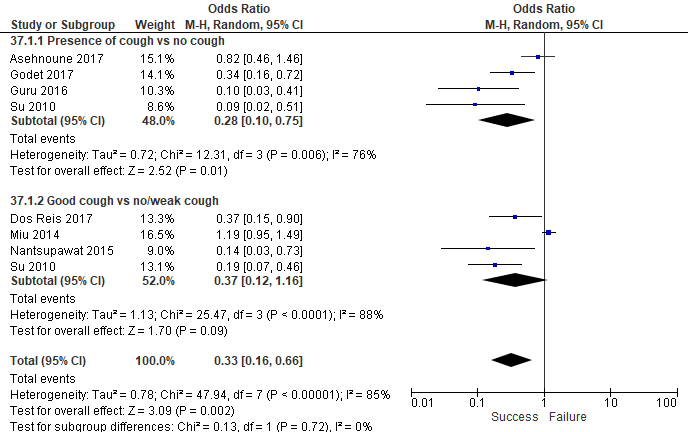


1. Cough peak flow


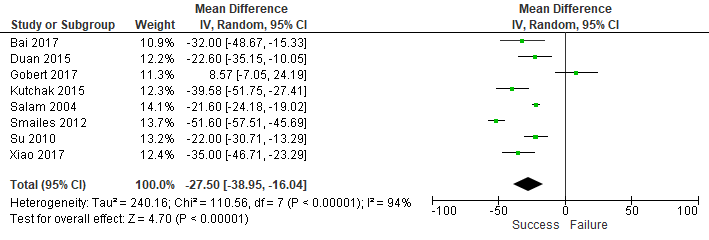


1. Duration of mechanical ventilation before extubation


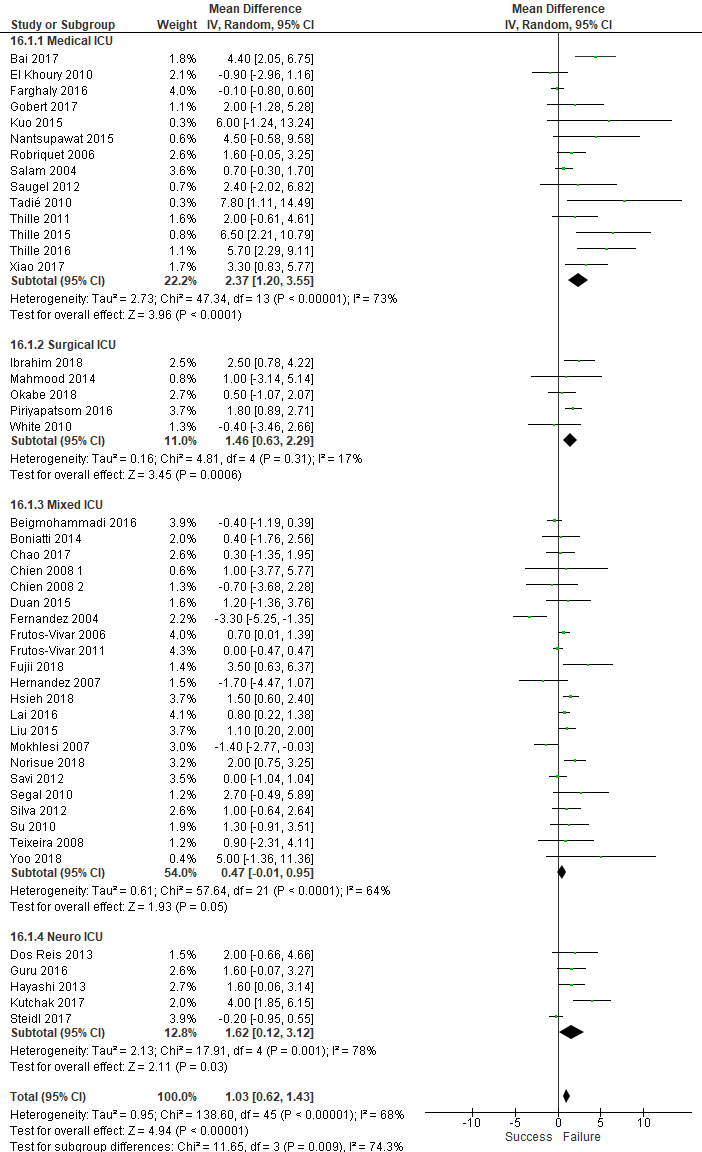


1. GCS at extubation


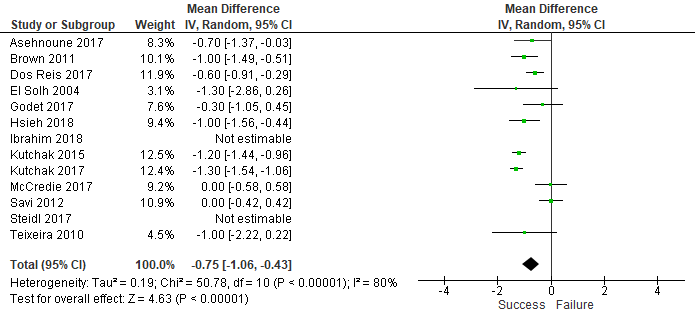


1. Heart rate before extubation


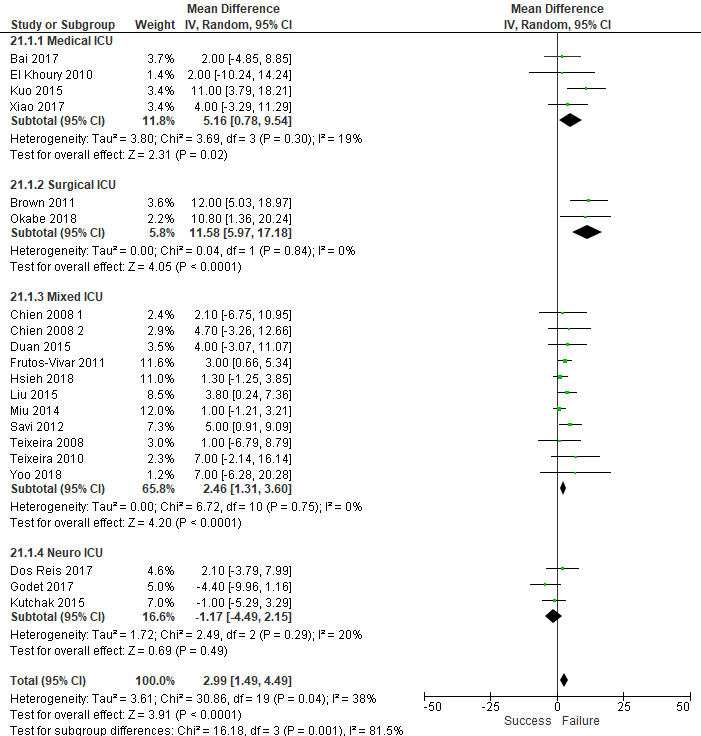


1. Hemoglobin on the day of extubation


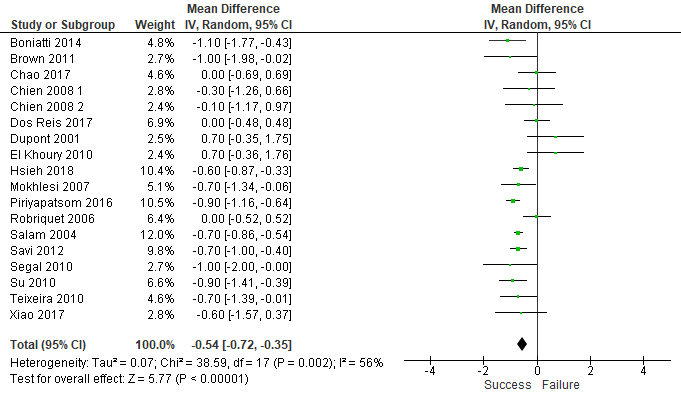


1. History of cardiac disease


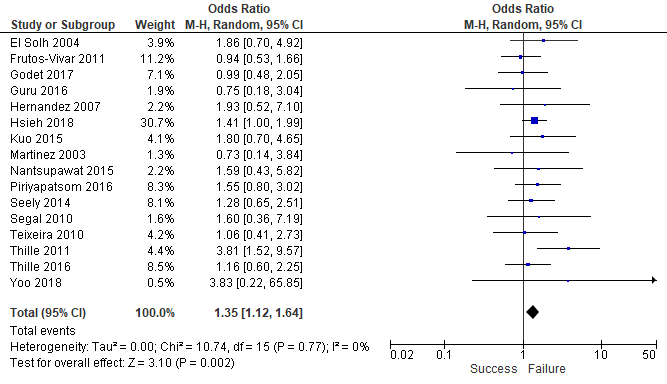


1. History of respiratory disease


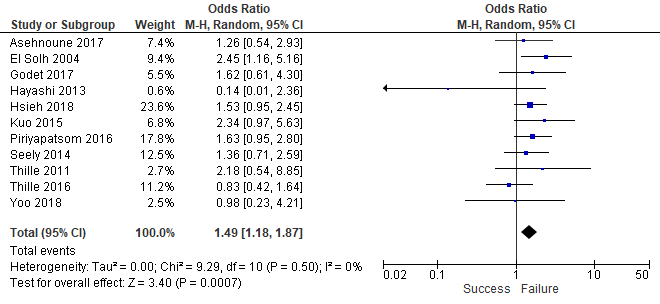


1. Maximal expiratory pressure


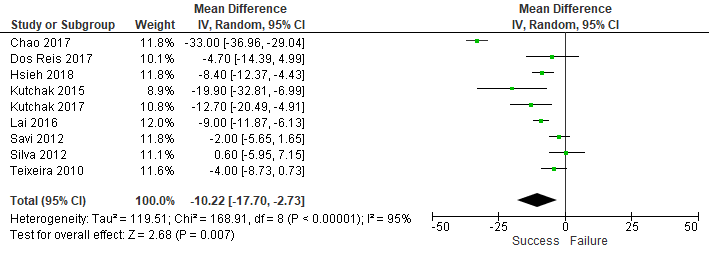


1. Presence of moderate to abundant secretions


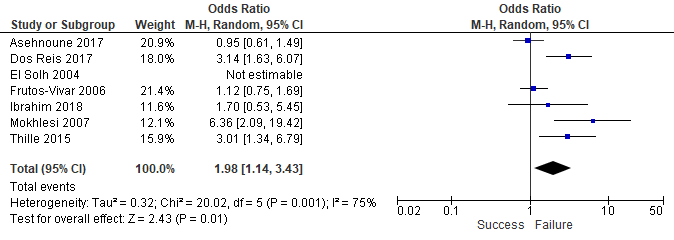


1. Negative inspiratory force


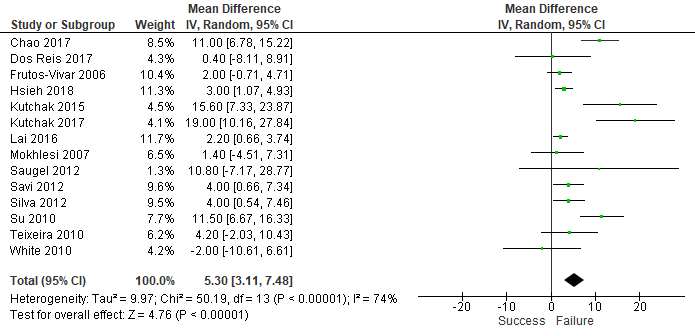


1. PaO_2_ before extubation


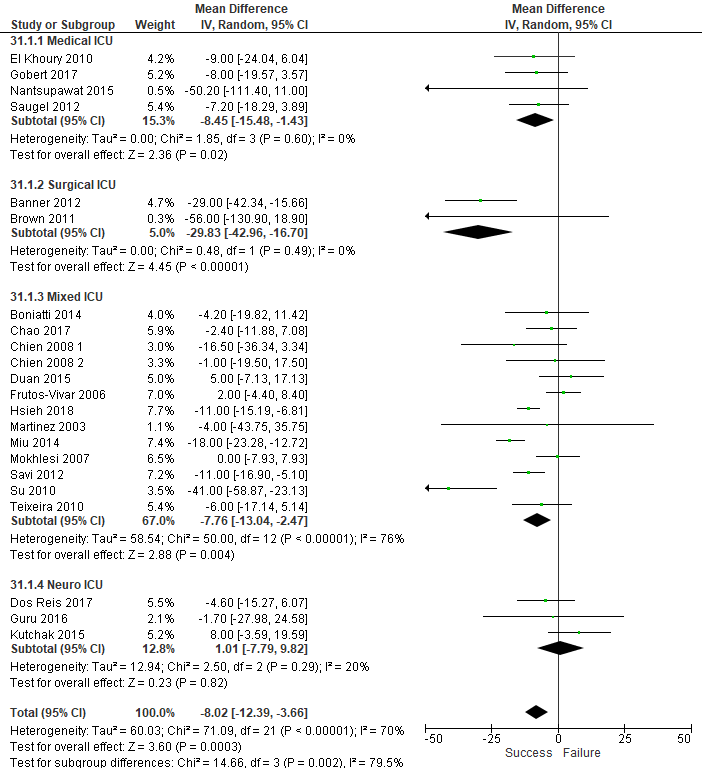


1. PaO_2_/FiO_2_ before extubation


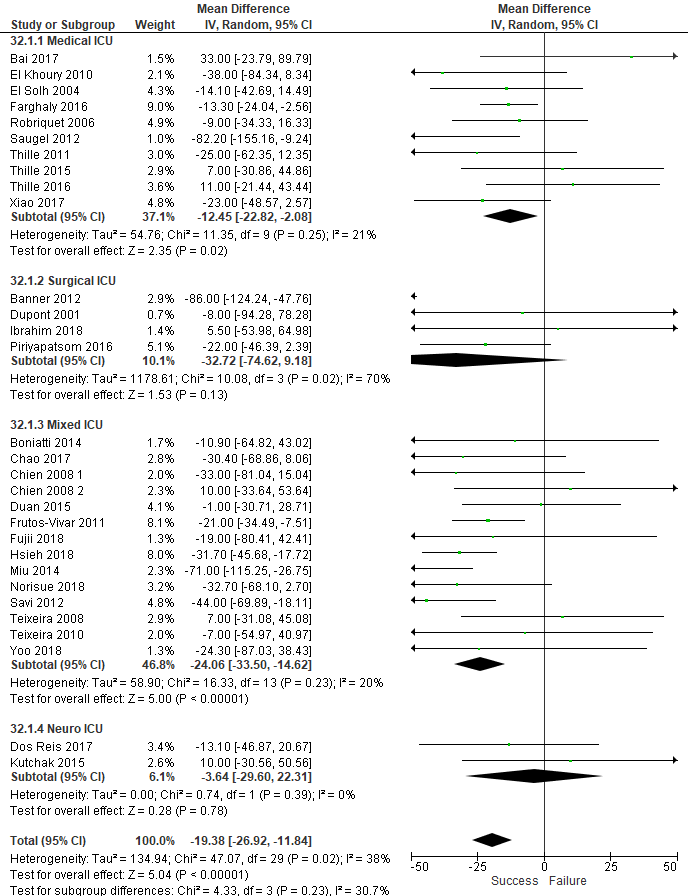


1. Pneumonia


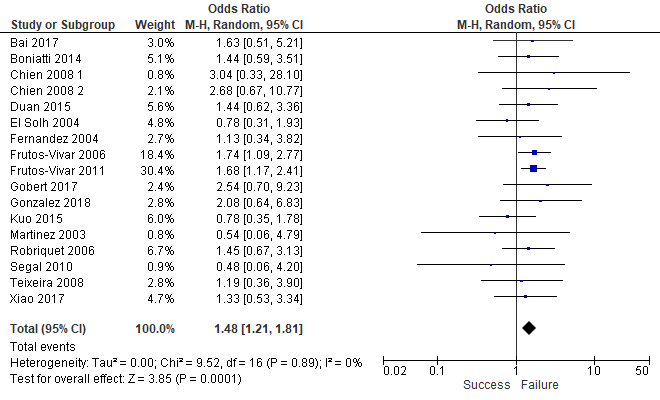


1. Respiratory rate before extubation


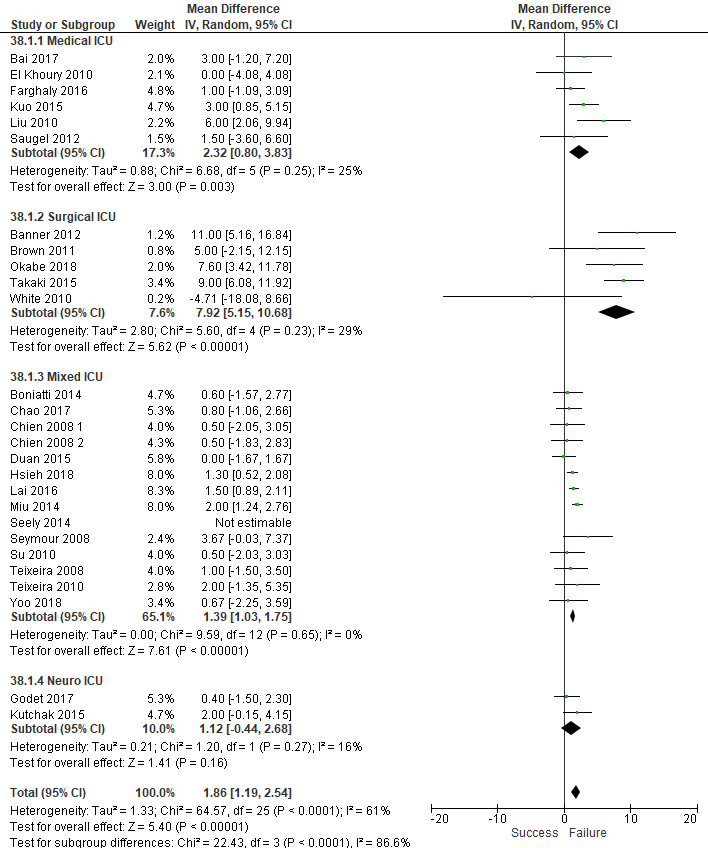


1. RSBI


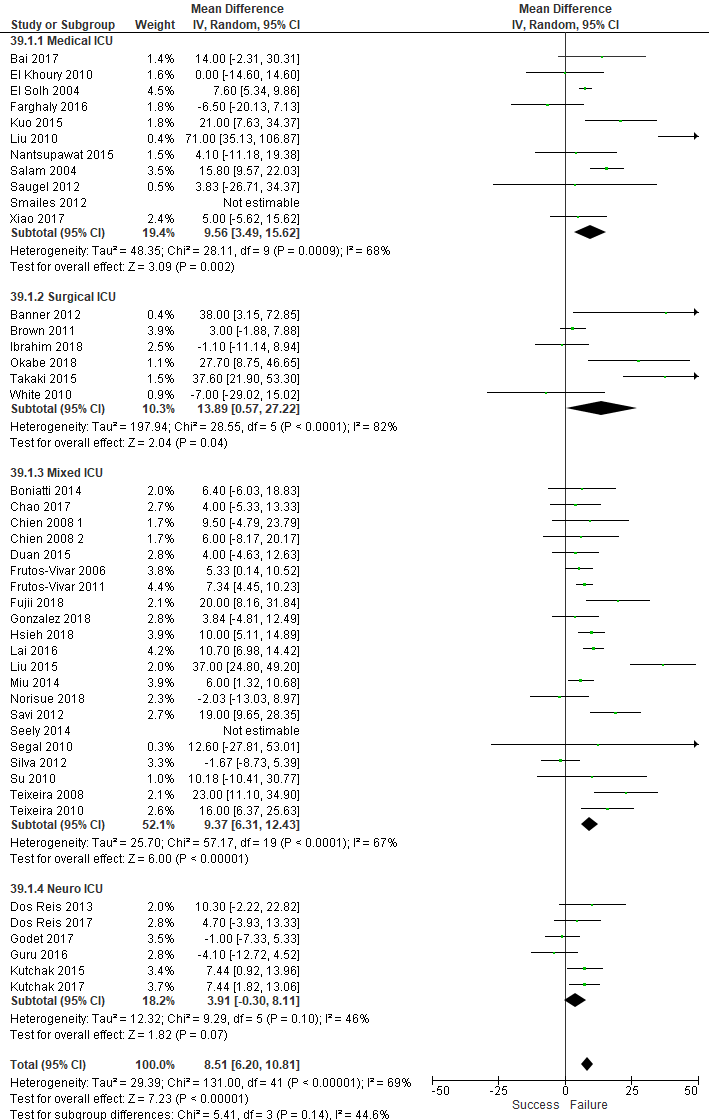


1. RSBI after 1 minute of a spontaneous breathing trial


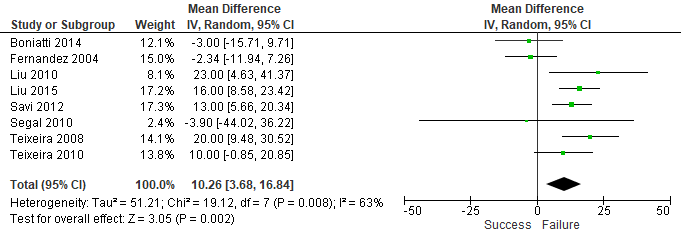


1. SaO_2_ before extubation


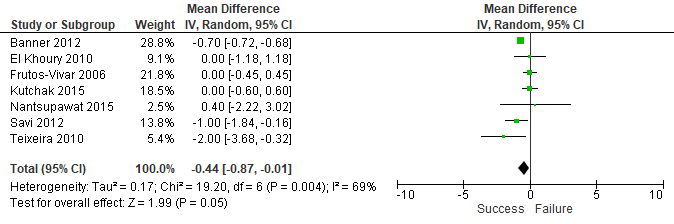


1. SAPS II


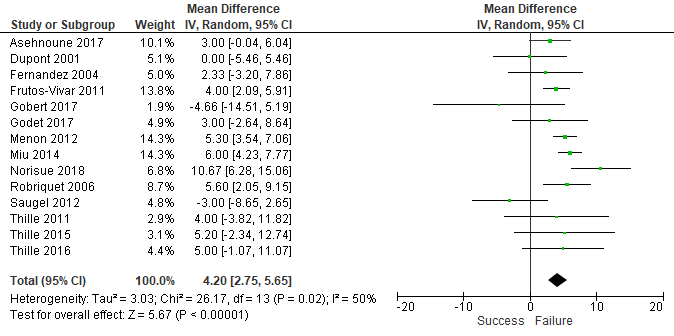


1. Tidal volume before extubation


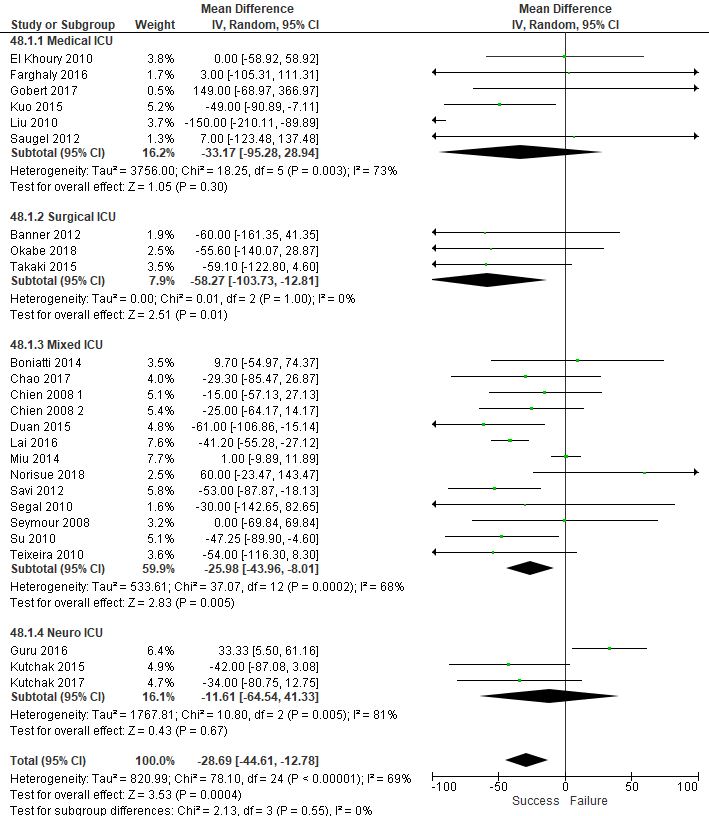


**Additional table S2.** Subgroup analyses by intensive care unit type.

| Variable | N (%) | | ICU type | n (%) | | Effect estimate (95% IC), p value | |
| --- | --- | --- | --- | --- | --- | --- | --- |
| Acute heart failure | 11 (16%) | | Medical | 3 | | 1.16 (0.56 to 2.43 ), p= 0.69 | |
|  |  | | Mixed | 8 | | 1.47 (0.98 to 2.19 ), p= 0.06 | |
| Acute respiratory failure | 7 (10%) | | Mixed | 3 | | 0.93 (0.5 to 1.73 ), p= 0.83 | |
|  |  | | Surgical | 2 | | 2.78 (1.28 to 6.05 ), p= 0.01 | |
|  |  | | Medical | 2 | | 1.51 (0.46 to 4.96 ), p= 0.49 | |
| Age | 59 (88%) | | Medical | 15 | | 3.69 (1.44 to 5.94 ), p= 0.001 | |
|  |  | | Surgical | 7 | | 2.71 (-0.44 to 5.86 ), p= 0.09 | |
|  |  | | Mixed | 26 | | 3.7 (2.78 to 4.62 ), p= <0.001 | |
|  |  | | Neurological | 11 | | 3.38 (1.49 to 5.28 ), p= <0.001 | |
| Albumin | 9 (13%) | | Mixed | 5 | | -0.09 (-0.23 to 0.06 ), p= 0.24 | |
|  |  | | Surgical | 1 | | -3 (-5.49 to -0.51 ), p= 0.02 | |
|  |  | | Medical | 1 | | -0.3 (-0.99 to 0.39 ), p= 0.4 | |
|  |  | | Other | 2 | | -2.56 (-3.9 to -1.22 ), p= <0.001 | |
| APACHE II score | 33 (49%) | | Medical | 5 | | 1.22 (-1.33 to 3.77 ), p= 0.35 | |
|  |  | | Surgical | 4 | | 0.94 (-2.08 to 3.97 ), p= 0.54 | |
|  |  | | Mixed | 21 | | 1.8 (1.03 to 2.57 ), p= <0.001 | |
|  |  | | Neurological | 3 | | 1.98 (0.86 to 3.11 ), p= 0.001 | |
| ARDS | 9 (13%) | | Mixed | 6 | | 1.12 (0.7 to 1.77 ), p= 0.64 | |
|  |  | | Medical | 1 | | 0.68 (0.14 to 3.4 ), p= 0.64 | |
|  |  | | Other | 2 | | 1.3 (0.26 to 6.43 ), p= 0.75 | |
| BMI | 13 (19%) | | Mixed | 5 | | -0.53 (-1 to -0.05 ), p= 0.03 | |
|  |  | | Medical | 3 | | -2.44 (-5.05 to 0.17 ), p= 0.07 | |
|  |  | | Surgical | 2 | | -0.92 (-2.53 to 0.68 ), p= 0.26 | |
|  |  | | Other | 1 | | 1.6 (-0.34 to 3.54 ), p= 0.11 | |
|  |  | | Neurological | 2 | | -0.22 (-1.06 to 0.62 ), p= 0.61 | |
| History of cardiac disease | 16 (24%) | | Mixed | 8 | | 1.27 (0.99 to 1.63 ), p= 0.06 | |
|  |  | | Medical | 4 | | 1.84 (1.1 to 3.07 ), p= 0.02 | |
|  |  | | Surgical | 1 | | 1.55 (0.8 to 3.02 ), p= 0.2 | |
|  |  | | Other | 1 | | 1.59 (0.43 to 5.82 ), p= 0.49 | |
|  |  | | Neurological | 2 | | 0.94 (0.49 to 1.78 ), p= 0.84 | |
| History of respiratory disease | 11 (16%) | | Mixed | 3 | | 1.43 (0.99 to 2.07 ), p= 0.06 | |
|  |  | | Medical | 5 | | 1.65 (1.03 to 2.65 ), p= 0.04 | |
|  |  | | Surgical | 1 | | 1.63 (0.95 to 2.8 ), p= 0.08 | |
|  |  | | Neurological | 2 | | 0.61 (0.07 to 5.36 ), p= 0.66 | |
| Coma | 8 (12%) | | Mixed | 5 | | 0.76 (0.55 to 1.05 ), p= 0.1 | |
|  |  | | Medical | 2 | | 0.71 (0.32 to 1.59 ), p= 0.4 | |
|  |  | | Surgical | 1 | | 1.08 (0.29 to 4.01 ), p= 0.9 | |
| COPD | 12 (18%) | | Mixed | 8 | | 1.62 (1.15 to 2.28 ), p= 0.01 | |
|  |  | | Medical | 1 | | 2.83 (0.25 to 31.74 ), p= 0.4 | |
|  |  | | Surgical | 1 | | 2.4 (0.21 to 27.59 ), p= 0.48 | |
|  |  | | Other | 1 | | 1.43 (0.42 to 4.91 ), p= 0.57 | |
|  |  | | Neurological | 1 | | 0.71 (0.07 to 6.76 ), p= 0.76 | |
| COPD exacerbation | | 17 (25%) | Mixed | | 12 | 1.14 (0.87 to 1.5 ), p= 0.3 |  |
|  |  |  | Medical | | 3 | 2.66 (1.51 to 4.68 ), p= 0.001 |  |
|  |  |  | Other | | 2 | 1.17 (0.67 to 2.02 ), p= 0.59 |  |
| Cough versus No or weak cough | | 7 (10%) | Mixed | | 2 | 0.38 (0.03 to 4.59 ), p= 0.45 |  |
|  |  |  | Neurological | | 3 | 0.38 (0.18 to 0.79 ), p= 0.009 |  |
|  |  |  | Other | | 1 | 0.14 (0.03 to 0.73 ), p= 0.02 |  |
| Creatinine | | 9 (13%) | Mixed | | 5 | 0.18 (-0.1 to 0.46 ), p= 0.2 |  |
|  |  |  | Medical | | 1 | -0.3 (-0.81 to 0.21 ), p= 0.24 |  |
|  |  |  | Surgical | | 2 | 0.24 (-0.25 to 0.73 ), p= 0.35 |  |
|  |  |  | Other | | 1 | 0 (-0.16 to 0.16 ), p= 1 |  |
| Diastolic blood pressure pre extubation | | 9 (13%) | Mixed | | 4 | -1.89 (-4.63 to 0.84 ), p= 0.18 |  |
|  |  |  | Medical | | 1 | -1 (-8.15 to 6.15 ), p= 0.78 |  |
|  |  |  | Neurological | | 2 | 1.1 (-1.73 to 3.93 ), p= 0.45 |  |
|  |  |  | Other | | 2 | -2.15 (-5.66 to 1.36 ), p= 0.23 |  |
| Diabetes | | 14 (21%) | Mixed | | 9 | 1.1 (0.75 to 1.63 ), p= 0.62 |  |
|  |  |  | Surgical | | 1 | 0.56 (0.1 to 3.23 ), p= 0.51 |  |
|  |  |  | Neurological | | 3 | 1.54 (0.91 to 2.61 ), p= 0.11 |  |
|  |  |  | Other | | 1 | 1.71 (0.47 to 6.28 ), p= 0.42 |  |
| Duration of MV before extubation | | 46 (69%) | Medical | | 14 | 2.37 (1.2 to 3.55 ), p= <0.001 |  |
|  |  |  | Surgical | | 5 | 1.46 (0.63 to 2.29 ), p= 0.001 |  |
|  |  |  | Mixed | | 22 | 0.47 (-0.01 to 0.95 ), p= 0.05 |  |
|  |  |  | Neuro | | 5 | 1.62 (0.12 to 3.12 ), p= 0.03 |  |
| GCS at extubation | | 12 (18%) | Mixed | | 3 | -0.59 (-1.38 to 0.2 ), p= 0.14 |  |
|  |  |  | Medical | | 1 | -1.3 (-2.86 to 0.26 ), p= 0.1 |  |
|  |  |  | Surgical | | 1 | -1 (-1.49 to -0.51 ), p= <0.001 |  |
|  |  |  | Neurological | | 7 | -0.75 (-1.14 to -0.37 ), p= <0.001 |  |
| GCS upon admission | | 12 (18%) | Mixed | | 4 | -0.2 (-0.58 to 0.18 ), p= 0.3 |  |
|  |  |  | Surgical | | 1 | -0.5 (-1.33 to 0.33 ), p= 0.24 |  |
|  |  |  | Neurological | | 7 | -0.33 (-0.88 to 0.22 ), p= 0.24 |  |
| Heart rate pre extubation | | 20 (30%) | Medical | | 4 | 5.16 (0.78 to 9.54 ), p= 0.02 |  |
|  |  |  | Surgical | | 2 | 11.58 (5.97 to 17.18 ), p= <0.001 |  |
|  |  |  | Mixed | | 11 | 2.46 (1.31 to 3.6 ), p= <0.001 |  |
|  |  |  | Neurological | | 3 | -1.17 (-4.49 to 2.15 ), p= 0.49 |  |
| Hemoglobin | | 18 (27%) | Mixed | | 10 | -0.66 (-0.81 to -0.5 ), p= <0.001 |  |
|  |  |  | Medical | | 2 | -0.1 (-1.46 to 1.26 ), p= 0.88 |  |
|  |  |  | Neurological | | 1 | 0 (-0.48 to 0.48 ), p= 1 |  |
|  |  |  | Other | | 2 | -0.15 (-0.67 to 0.36 ), p= 0.56 |  |
|  |  |  | Surgical | | 3 | -0.48 (-1.39 to 0.42 ), p= 0.3 |  |
| History of hypertension | | 8 (12%) | Mixed | | 5 | 1.05 (0.73 to 1.52 ), p= 0.79 |  |
|  |  |  | Surgical | | 1 | 0.96 (0.27 to 3.46 ), p= 0.96 |  |
|  |  |  | Neurological | | 1 | 1.15 (0.37 to 3.57 ), p= 0.81 |  |
|  |  |  | Other | | 1 | 3.44 (0.42 to 28.29 ), p= 0.25 |  |
| Males | | 52 (78%) | Medical | | 12 | 0.95 (0.53 to 1.68 ), p= 0.85 |  |
|  |  |  | Surgical | | 5 | 1.16 (0.56 to 2.42 ), p= 0.69 |  |
|  |  |  | Mixed | | 25 | 0.94 (0.77 to 1.13 ), p= 0.49 |  |
|  |  |  | Neuro | | 10 | 0.7 (0.43 to 1.13 ), p= 0.14 |  |
| Mean arterial pressure pre extubation | | 7 (10%) | Mixed | | 5 | -1 (-2.44 to 0.44 ), p= 0.18 |  |
|  |  |  | Neurological | | 1 | -0.2 (-6.69 to 6.29 ), p= 0.95 |  |
|  |  |  | Other | | 1 | -0.2 (-15.7 to 15.3 ), p= 0.98 |  |
| Maximal Expiratory Pressure | | 9 (13%) | Mixed | | 6 | -9.39 (-18.81 to 0.03 ), p= 0.05 |  |
|  |  |  | neurological | | 3 | -11.7 (-19.36 to -4.04 ), p= 0.003 |  |
| Minute ventilation pre extubation | | 17 (25%) | Mixed | | 12 | -0.08 (-0.49 to 0.34 ), p= 0.71 |  |
|  |  |  | Medical | | 3 | -0.1 (-0.77 to 0.56 ), p= 0.76 |  |
|  |  |  | Surgical | | 1 | 2.7 (0.86 to 4.54 ), p= 0.004 |  |
| Neurologic diagnosis | | 9 (13%) | Mixed | | 6 | 1.01 (0.59 to 1.71 ), p= 0.98 |  |
|  |  |  | Medical | | 3 | 1.81 (0.78 to 4.19 ), p= 0.17 |  |
| Negative inspiratory force | | 14 (21%) | Mixed | | 9 | 4.46 (2.49 to 6.43 ), p= < 0.001 |  |
|  |  |  | Medical | | 1 | 10.8 (-7.17 to 28.77 ), p= 0.24 |  |
|  |  |  | Surgical | | 1 | -2 (-10.61 to 6.61 ), p= 0.65 |  |
|  |  |  | Neurological | | 3 | 11.65 (0.49 to 22.8 ), p= 0.04 |  |
| FiO_2_ during SBT | | 11 (16%) | Mixed | | 5 | 0.01 (0.00, 0.01), p=0.001 |  |
|  |  |  | Surgical | | 3 | -0.01 (-0.04, 0.02), p= 0.6 |  |
|  |  |  | Neurological | | 3 | 0.00 (-0.00, 0.00), p=1 |  |
| PaCO_2_ pre extubation | | 34 (51%) | Medical | | 11 | 1.06 (-0.92 to 3.05 ), p= 0.29 |  |
|  |  |  | Surgical | | 3 | 0.65 (-3.39 to 4.69 ), p= 0.75 |  |
|  |  |  | Mixed | | 17 | 1.08 (-0.06 to 2.22 ), p= 0.06 |  |
|  |  |  | Neurological | | 3 | -0.51 (-1.71 to 0.68 ), p= 0.4 |  |
| PaO_2_ pre extubation | | 22 (33%) | Medical | | 4 | -8.45 (-15.48 to -1.43 ), p= 0.02 |  |
|  |  |  | Surgical | | 2 | -29.83 (-42.96 to -16.7 ), p= <0.001 |  |
|  |  |  | Mixed | | 13 | -7.76 (-13.04 to -2.47 ), p= 0.004 |  |
|  |  |  | Neurological | | 3 | 1.01 (-7.79 to 9.82 ), p= 0.82 |  |
| PaO_2_/FiO_2_ pre extubation | | 30 (45%) | Medical | | 10 | -12.45 (-22.82 to -2.08 ), p= 0.02 |  |
|  |  |  | Surgical | | 4 | -32.72 (-74.62 to 9.18 ), p= 0.13 |  |
|  |  |  | Mixed | | 14 | -24.06 (-33.5 to -14.62 ), p= <0.001 |  |
|  |  |  | Neurological | | 2 | -3.64 (-29.6 to 22.31 ), p= 0.78 |  |
| pH pre extubation | | 27 (40%) | Medical | | 10 | 0 (-0.01 to 0.01 ), p= 0.87 |  |
|  |  |  | Surgical | | 2 | -0.01 (-0.03 to 0.01 ), p= 0.42 |  |
|  |  |  | Mixed | | 13 | -0.01 (-0.01 to 0 ), p= 0.23 |  |
|  |  |  | Neurological | | 2 | -0.01 (-0.02 to 0 ), p= 0.14 |  |
| PEEP during SBT | | 11 (16%) | Mixed | | 6 | 0.04 (-0.02 to 0.09 ), p= 0.19 |  |
|  |  |  | Surgical | | 2 | -0.19 (-0.91 to 0.53 ), p= 0.61 |  |
|  |  |  | Neurological | | 3 | 0.1 (-0.02 to 0.22 ), p= 0.1 |  |
| Pneumonia | | 17 (25%) | Mixed | | 11 | 1.61 (1.27 to 2.04 ), p= <0.001 |  |
|  |  |  | Medical | | 3 | 1 (0.53 to 1.91 ), p= 1 |  |
|  |  |  | Other | | 3 | 1.44 (0.85 to 2.45 ), p= 0.17 |  |
| Respiratory rate pre extubation | | 27 (40%) | Medical | | 6 | 2.32 (0.8 to 3.83 ), p= 0.003 |  |
|  |  |  | Surgical | | 5 | 7.92 (5.15 to 10.68 ), p= <0.001 |  |
|  |  |  | Mixed | | 14 | 1.39 (1.03 to 1.75 ), p= <0.001 |  |
|  |  |  | Neurological | | 2 | 1.12 (-0.44 to 2.68 ), p= 0.16 |  |
| RSBI | | 44 (66%) | Medical | | 11 | 9.56 (3.49 to 15.62 ), p= 0.002 |  |
|  |  |  | Surgical | | 6 | 13.89 (0.57 to 27.22 ), p= 0.04 |  |
|  |  |  | Mixed | | 21 | 9.37 (6.31 to 12.43 ), p= <0.001 |  |
|  |  |  | Neurological | | 6 | 3.91 (-0.3 to 8.11 ), p= 0.07 |  |
| RSBI, 1 min SBT | | 8 (12%) | Mixed | | 7 | 9.14 (2.28 to 15.99 ), p= 0.009 |  |
|  | |  | Medical | | 1 | 23 (4.63 to 41.37 ), p= 0.01 |  |
| SaO_2_ pre extubation | | 7 (10%) | Mixed | | 3 | -0.78 (-1.81 to 0.25 ), p= 0.14 |  |
|  |  |  | Medical | | 1 | 0 (-1.18 to 1.18 ), p= 1 |  |
|  |  |  | Surgical | | 1 | -0.7 (-0.72 to -0.68 ), p= <0.001 |  |
|  |  |  | Neurological | | 1 | 0 (-0.6 to 0.6 ), p= 1 |  |
|  |  |  | Other | | 1 | 0.4 (-2.22 to 3.02 ), p= 0.76 |  |
| SAPS II | | 15 (22%) | Mixed | | 6 | 5.51 (3.81 to 7.2 ), p= <0.001 |  |
|  |  |  | Medical | | 5 | 1.52 (-2.56 to 5.6 ), p= 0.47 |  |
|  |  |  | Surgical | | 1 | 0 (-5.46 to 5.46 ), p= 1 |  |
|  |  |  | Neurological | | 2 | 3 (0.32 to 5.68 ), p= 0.03 |  |
|  |  |  | Other | | 1 | 5.6 (2.05 to 9.15 ), p= 0.002 |  |
| Presence of moderate to abundant secretions | | 7 (10%) | Mixed | | 2 | 2.47 (0.45 to 13.4 ), p= 0.3 |  |
|  |  |  | Medical | | 2 | 3.01 (1.34 to 6.79 ), p= 0.01 |  |
|  |  |  | Surgical | | 1 | 1.7 (0.53 to 5.45 ), p= 0.37 |  |
|  |  |  | Neurological | | 2 | 1.69 (0.52 to 5.43 ), p= 0.38 |  |
| Tidal volume pre extubation | | 25 (37%) | Medical | | 6 | -33.17 (-95.28 to 28.94 ), p= 0.3 |  |
|  |  |  | Surgical | | 3 | -58.27 (-103.73 to -12.81 ), p= 0.01 |  |
|  |  |  | Mixed | | 12 | -25.98 (-43.96 to -8.01 ), p= 0.005 |  |
|  |  |  | Neurological | | 3 | -11.61 (-64.54 to 41.33 ), p= 0.67 |  |
| Cough peak flow | | 8 (12%) | Mixed | | 2 | -22.19 (-29.35 to -15.04 ), p= <0.001 |  |
|  |  |  | Medical | | 2 | -7.54 (-37.04 to 21.96 ), p= 0.62 |  |
|  |  |  | Other | | 3 | -40.89 (-54.67 to -27.11 ), p= <0.001 |  |
|  |  |  | Neurological | | 1 | -39.58 (-51.75 to -27.41 ), p= <0.001 |  |
| Postoperative respiratory failure | | 7 (10%) | Mixed | | 5 | 0.94 (0.66 to 1.32 ), p= 0.71 |  |
|  |  |  | Surgical | | 1 | 0.52 (0.14 to 1.92 ), p= 0.33 |  |
|  |  |  | Medical | | 1 | 2.25 (0.84 to 6.03 ), p= 0.11 |  |
| Systolic blood pressure pre extubation | | 12 (18%) | Mixed | | 6 | -0.2 (-2.56 to 2.15 ), p= 0.87 |  |
|  |  |  | Medical | | 2 | 3.49 (-4.13 to 11.1 ), p= 0.37 |  |
|  |  |  | Surgical | | 1 | -2 (-10.8 to 6.8 ), p= 0.66 |  |
|  |  |  | Other | | 2 | -3.24 (-10.15 to 3.66 ), p= 0.36 |  |
|  |  |  | Neurological | | 1 | 2 (-4.52 to 8.52 ), p= 0.55 |  |
| Sepsis | | 7 (10%) | Mixed | | 6 | 1.17 (0.92 to 1.49 ), p= 0.2 |  |
|  |  |  | Medical | | 1 | 1.01 (0.29 to 3.58 ), p= 0.99 |  |
| Shock | | 8 (12%) | Mixed | | 4 | 0.72 (0.29 to 1.76 ), p= 0.47 |  |
|  |  |  | Medical | | 4 | 0.99 (0.41 to 2.39 ), p= 0.99 |  |
| Steroids | | 7 (10%) | Mixed | | 3 | 0.96 (0.44 to 2.13 ), p= 0.93 |  |
|  |  |  | Medical | | 1 | 0.62 (0.13 to 2.93 ), p= 0.55 |  |
|  |  |  | Surgical | | 1 | 0.85 (0.46 to 1.58 ), p= 0.62 |  |
|  |  |  | Neurological | | 1 | 0.28 (0.08 to 0.93 ), p= 0.04 |  |
|  |  |  | Other | | 1 | 1.17 (0.59 to 2.29 ), p= 0.66 |  |
| Trauma | | 7 (10%) | Mixed | | 6 | 0.83 (0.62 to 1.13 ), p= 0.23 |  |
|  |  |  | Surgical | | 1 | 0.55 (0.18 to 1.72 ), p= 0.3 |  |

N: Number of studies; n: Number of studies in ICU subgroup ; ARDS: acute respiratory distress syndrom; BMI: body mass index; COPD: chronic obstructive pulmonary disease; MV: mechanical ventilation; GCS: glasgow coma scale; SBT: spontaneous breathing trial; PEEP: positiv end expiratory pressure; RSBI: Rapid shallow breathing index; SAPS II: simplifed acute Physiology Score II.

**Additional figure S2.** Heatmap of natural log transformation of odd ratios (LnOR) for extubation failure, according to ICU type.


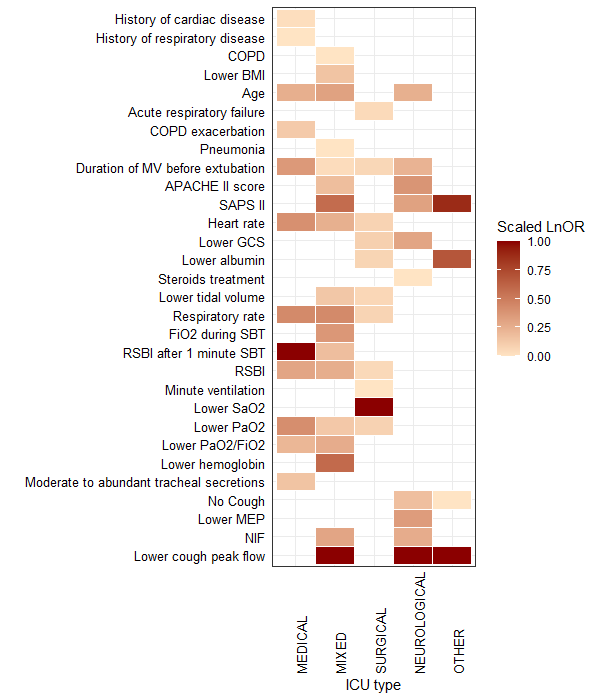


Natural log transformation of odd ratios (LnOR) derived from crude OR (for binary variables) and from standardized mean differences (for continuous variables), summarizing the effect of 30 variables significantly associated with extubation outcome in at least one intensive care unit (ICU) type. LnOR factors were scaled to adjust for extreme values. White boxes represent missing or nonsignificant values.

**Additional table S3:** Sensitivity analysis focusing on studies defining extubation failure as death or reintubation, whatever the delay.

| Variables | Statistical significance  after sensitivity analysis |
| --- | --- |
| Acute heart failure | yes |
| Age | yes |
| APACHE II score | yes |
| BMI | no |
| COPD | yes |
| COPD exacerbation | no |
| Cough* | yes |
| Cough peak flow | yes |
| Duration of mechanical ventilation before extubation | yes |
| GCS at extubation | yes |
| Heart rate before extubation | yes |
| Hemoglobin on the day of extubation | yes |
| History of cardiac disease | yes |
| History of respiratory disease | yes |
| Maximal expiratory pressure | yes |
| Negative inspiratory force | yes |
| PaO_2_ before extubation | yes |
| PaO_2_/FiO_2_ before extubation | yes |
| Pneumonia | yes |
| Presence of moderate to abundant secretions | yes |
| Respiratory rate before extubation | yes |
| RSBI before extubation | yes |
| RSBI after 1 minute of a spontaneous breathing trial | no |
| SaO_2_ before extubation | yes |
| SAPS II | yes |
| Tidal volume before extubation | yes |

BMI: body mass index; COPD: chronic obstructive pulmonary disease; GCS: glasgow coma scale; RSBI: Rapid shallow breathing index; SAPS II: simplifed acute Physiology Score II.

**Additional table S4.** Sensitivity analysis focusing on studies defining extubation failure at 48 hours.

| Variables | Statistical significance  after sensitivity analysis |
| --- | --- |
| Acute heart failure | yes |
| Age | yes |
| APACHE II score | yes |
| BMI | no |
| COPD | yes |
| COPD exacerbation | no |
| Cough* | no |
| Cough peak flow | no |
| Duration of mechanical ventilation before extubation | no |
| GCS at extubation | yes |
| Heart rate before extubation | yes |
| Hemoglobin on the day of extubation | yes |
| History of cardiac disease | no |
| History of respiratory disease | no |
| Maximal expiratory pressure | yes |
| Negative inspiratory force | yes |
| PaO2 before extubation | yes |
| PaO2/FiO2 before extubation | yes |
| Pneumonia | yes |
| Presence of moderate to abundant secretions | no |
| Respiratory rate before extubation | yes |
| RSBI before extubation | yes |
| RSBI after 1 minute of a spontaneous breathing trial | no |
| SaO2 before extubation | no |
| SAPS II | no |
| Tidal volume before extubation | yes |

*: cough was considered in only one study included in the sensitivity analysis.

BMI: body mass index; COPD: chronic obstructive pulmonary disease; GCS: glasgow coma scale; RSBI: Rapid shallow breathing index; SAPS II: simplifed acute Physiology Score II.

**Additional figure S3.** Forest plot for the 26 variables significantly associated with extubation failure, assessed by multivariate meta-analysis for multiple factors.


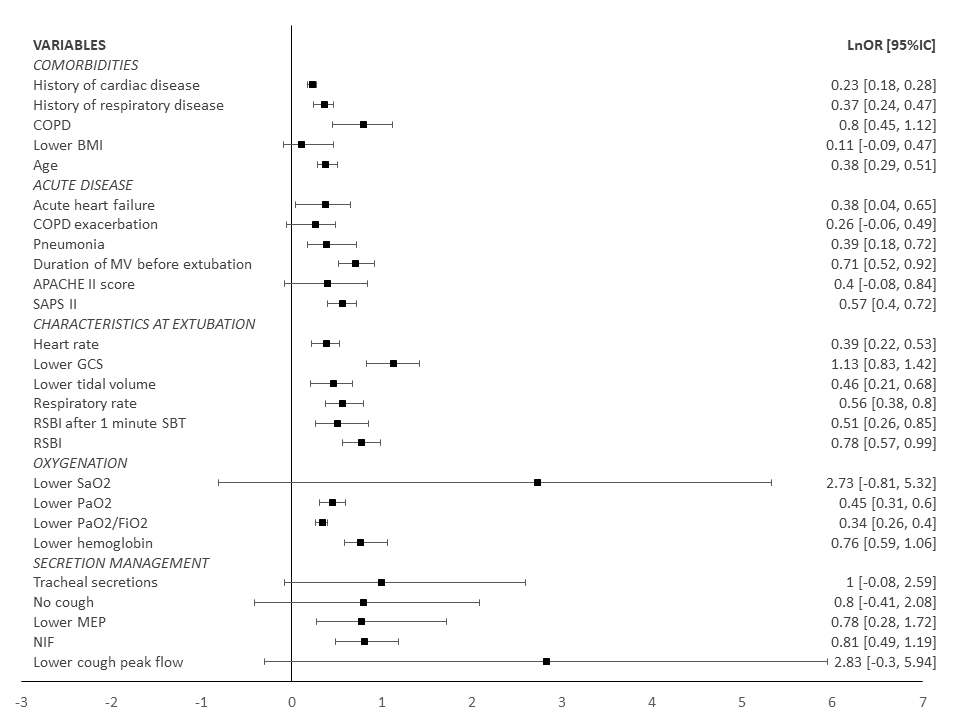


Effects are reported in natural log transformation of odd ratios (lnOR) derived from crude OR with 95% confidence interval margins (CI).

**Additional figure S4**. Individual risk of bias in the included studies.


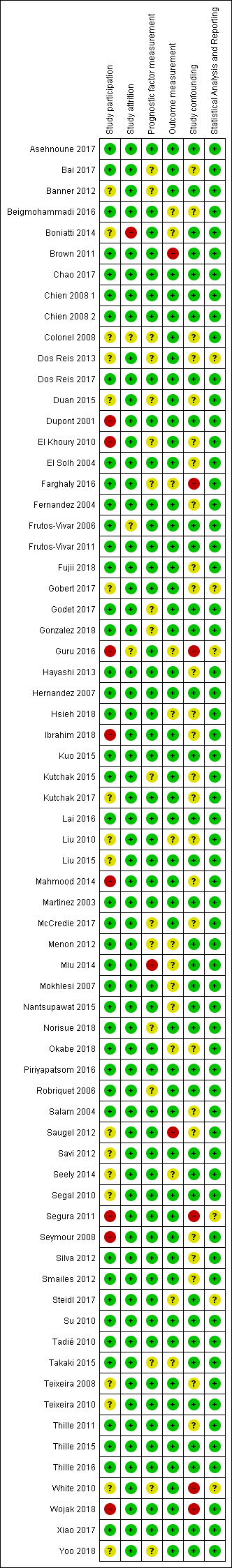

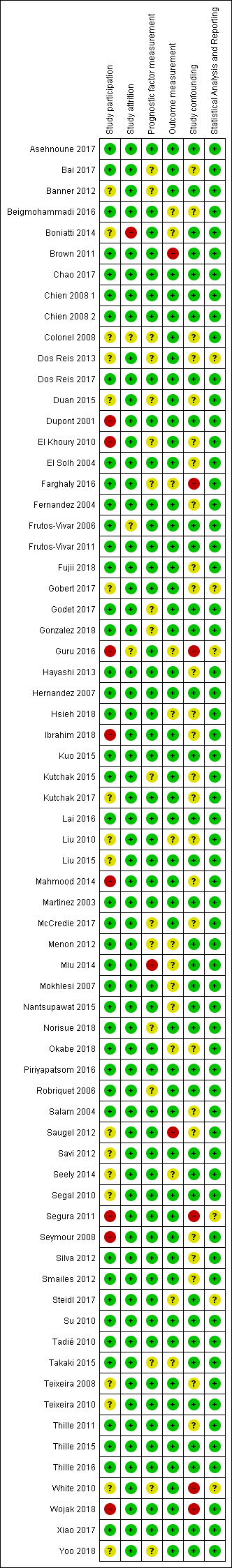

Supplement: Supplementary file 1 — Additional file 1: Table S1. Variables excluded because of missing data. Figure S1. Forest plots for variables statistically significantly associated with extubation failure. Table S2. Subgroup analyses by intensive care unit type. Figure S2. Heatmap of natural log transformation of odd ratios (LnOR) for extubation failure, according to ICU type. Table S3. Sensitivity analysis focusing on studies defining extubation failure as death or reintubation, whatever the delay. Table S4. Sensitivity analysis focusing on studies defining extubation failure at 48 h. Figure S3. Individual risk of bias in the included studies. Figure S4. Forest plot for the 26 variables significantly associated with extubation failure, assessed by multivariate meta-analysis for multiple factors. [file 13054_2021_3802_MOESM1_ESM.docx]
